# The Treatment of Fractures by Movement and Massage

**Published:** 1900-08-25

**Authors:** M. Lucas Championnière

**Affiliations:** Surgeon to the Hotel Dieu.


### Hospital Clinics and Medical Progress.

### THE TREATMENT OF FRACTURES BY MOVEMENT AND MASSAGE.

Summary of addresses given before the International Medical Congress in Paris by M. Lucas Championnière, Surgeon to the Hotel Dieu.

The question of employing massage and early movement in many cases of fracture which are commonly treated by immovable apparatus attracted a good deal of attention at the Paris Congress, where M. Lucas Championnière not only dealt with the subject in the surgical section, but also gave a demonstration at the hospital. The following resumé is condensed from what he said on these occasions.

By the treatment of fractures by massage and mobilisation one means the immediate application of movement to the injured limb, and one must be careful not to confound it with the secondary employment of massage to undo the mischief produced by the older forms of treatment nor with common rough rubbing, the violence of which might, and, indeed, often would, set up mischief and give rise to pain. Its efficacy depends on the fact that absolute immobility is not so conducive to repair as is a certain amount and degree of movement. Avoidance of violent exertion or muscular effort, such, that is, as might cause distortion, is of course desirable in the treatment of all fractures, but absolute quiescence, the complete immobility which is so often insisted on in the treatment of fractures, is an unnatural condition which is opposed to the proper carrying on of even the ordinary functions of nutrition, not to mention the repair of an injury, and the problem is how to get movement in the proper degree without leading to distortion and contracture. One may see in the admirable manner in which fractures of bone are sometimes repaired in the lower animals how injurious are on the one hand absolute immobility, and on the other excessive movement. A dog with a broken limb will often obtain the most perfect union if left alone on a mat, and allowed to lick, or, in other words, to massage the broken limb. On the other hand, an attempt to apply the surgeon's art to such an accident, and to treat it by the aid of splints, frequently leads to a bad result; while, again, if such an animal is allowed to make great efforts, as, for example, in going up and down stairs, we also are apt to get great distortion. Thus we see that while slight movement leads to cure, immobility leads to stiffness, and great movement to disaster.

The movement which is employed in the treatment of fractures must then be used methodically, and its dosage must be carefully regulated. One way of applying movement is by massage—soft and progressive, affecting the parts in the neighbourhood of the fracture but not touching the place itself—but movement is possible without massage, and it must be borne in mind that it is not merely in regard to the bone that movement is required. In the soft parts, even more than in the bone, movement promotes repair. So far back as the years 1879 and 1880 M. Lucas Championnière had made a beginning in this direction, and had reported cases in which he had given up immobilisation in the treatment of fractures. He had begun by those involving joints, and had gone on to deal in the same way with all varieties of fractures, and he found that if massage and movement were used from the beginning the osseous repair was more rapid and more solid than in cases treated by absolute rest and immobility. The pain disappeared very promptly, and while its disappearance might be taken as the criterion of good massage and properly-applied movement, the persistence of pain, on the other hand, indicated that the method was not being properly used. Contracture, again, disappeared rapidly, and this removal of contracture played, he thought, a principal rôle in producing the disappearance of certain deformities which are apt to occur in connection with fractures, for example, of the olecranon, the clavicle, the shoulder, &c. No treatment contended so successfully against contracture as massage and movement. Other points of importance were that the absorption of blood and other effusions was much more rapid than where the limb was kept fixed, that nerves and muscles did not atrophy, and that when the bone was united the treatment was finished; for when the limb was kept immovable it not infrequently happened that after the fracture was cured the patient had to undergo another special treatment with the object of getting rid of the resulting stiffness, and of restoring the utility of the limb. The treatment, however, must not be confounded with a pure and simple abandonment of apparatus of all kinds, or with an attempt to put the limb at once to its ordinary uses. Certain fractures, it was true, can always be mobilised and massaged, with complete suppression of immobilisation properly so-called; others, again, can sometimes be treated without apparatus, but only exceptionally so; while in others, in which apparatus is necessary, the use of massage and movement in combination with such apparatus helps to obtain good results. There are certain cases, moreover, in which it is enough to secure movement without using massage, such as those in which, in infants, there is a tendency to the production of an excessive amount of callus, and those in old people, in which there may be some doubt about the integrity of the veins.

The best idea of the extent to which it has been found desirable to use the treatment by massage, by movement, and by one or other of these processes combined with apparatus, is to take the actual cases treated during the past five years by Dr. Dragon, M. Championnière's assistant, for such cases at the Hotel Dieu. They are as follows: Fibula, 65 cases, almost exclusively without apparatus for immobilisation. Leg, 90 cases, of the superior epiphysis; 10 cases without apparatus; of the middle region, 38 cases, in all except two by a combination of apparatus and massage; through the malleoli for the most part by massage and movement and without apparatus. Femur, six cases, in which massage was used as a supplement to other treatment. Scapula, three cases, treated by massage exclusively. Clavicle, 64 cases, treated by massage exclusively. Humerus, 60 cases, 40 of the upper extremity, as far down as the deltoid insertion, treated exclusively by massage and without apparatus; eight of the lower extremity, treated exclusively by massage; 12 of the middle region, three of which were treated by massage without apparatus, all the rest having a combination of apparatus and massage. Olecranon, 20 cases by massage and mobilisation without apparatus. Forearm and wrist, 22 cases, treated by various combinations of apparatus and movement. Radius, 124 cases, treated almost exclusively by massage and movement. Bones of the hand and foot, seven cases, treated by massage and movement without apparatus. It must not be imagined, however, that the treatment so ably advocated by M. Lucas Championnière was accepted without contention. But one has to note that the opposition did not take the form of an advocacy of the use of apparatus, but came from those who maintained the much more advanced position that an incision should be made and the bones accurately replaced and sutured in their correct position. M. Paul Thiéry, of Paris, speaking before the surgical section, maintained that the only way of obtaining a perfect result was by osseous suture; and while admitting that it would be some time before such a treatment would be generally adopted, he maintained that when properly done osseous suture was absolutely harmless, that it constituted the operation of choice in simple fractures, and that where riding was present it was the only efficacious means of obtaining an ideal coaptation of the fragments.

